# A statistical approach to root system classification

**DOI:** 10.3389/fpls.2013.00292

**Published:** 2013-08-01

**Authors:** Gernot Bodner, Daniel Leitner, Alireza Nakhforoosh, Monika Sobotik, Karl Moder, Hans-Peter Kaul

**Affiliations:** ^1^Division of Agronomy, Department of Crop Sciences, University of Natural Resources and Life SciencesVienna, Austria; ^2^Computational Science Center, University of ViennaVienna, Austria; ^3^Institute of Plant SociologyKlagenfurt, Austria; ^4^Department of Landscape, Spatial and Infrastructure Sciences, Institute of Applied Statistics and Computing, University of Natural Resources and Life SciencesVienna, Austria

**Keywords:** root system diversity, classification, plant functional types, cluster analysis, root architecture model, taxonomy

## Abstract

Plant root systems have a key role in ecology and agronomy. In spite of fast increase in root studies, still there is no classification that allows distinguishing among distinctive characteristics within the diversity of rooting strategies. Our hypothesis is that a multivariate approach for “plant functional type” identification in ecology can be applied to the classification of root systems. The classification method presented is based on a data-defined statistical procedure without a priori decision on the classifiers. The study demonstrates that principal component based rooting types provide efficient and meaningful multi-trait classifiers. The classification method is exemplified with simulated root architectures and morphological field data. Simulated root architectures showed that morphological attributes with spatial distribution parameters capture most distinctive features within root system diversity. While developmental type (tap vs. shoot-borne systems) is a strong, but coarse classifier, topological traits provide the most detailed differentiation among distinctive groups. Adequacy of commonly available morphologic traits for classification is supported by field data. Rooting types emerging from measured data, mainly distinguished by diameter/weight and density dominated types. Similarity of root systems within distinctive groups was the joint result of phylogenetic relation and environmental as well as human selection pressure. We concluded that the data-define classification is appropriate for integration of knowledge obtained with different root measurement methods and at various scales. Currently root morphology is the most promising basis for classification due to widely used common measurement protocols. To capture details of root diversity efforts in architectural measurement techniques are essential.

## Introduction

The evolution of root systems^1^ is closely related to plant colonization of terrestrial ecosystems (Kenrick, 2002; Sperry, 2003) where plants require roots for anchorage, water, and nutrient acquisition. Although roots are a key organ for plant adaptation to variable environments and therefore for biodiversity (Cornwell and Grubb, 2003), they have long been neglected in plant biology and agronomy. Recently, however, root systems receive increasing attention as a key for a “second green revolution” (Lynch, 2007; Gewin, 2010) leading to more resource efficient plants.

A proper characterization of root system diversity is essential for various purposes, such as crop improvement, prediction of changes in species distribution under global change, or exploration of root functions in the carbon cycle. To capture diversity, a classification scheme for root systems is needed. It is a major shortcoming that to-date we lack such a scheme and that only few recent contributions try to fill this gap (e.g., Zobel and Waisel, 2010). Most botanical textbooks do not go beyond a very general developmental distinction between tap and fibrous root systems, concentrating more on specialized morphological adaptations (e.g., haustorial roots, storage roots) that occur in certain species (Bresinsky et al., 2008).

An explicit proposal of root system classification was presented by Fitter (1987) based on root topology. Fitter (1987) derived a topological index as a measure of functional diversity in resource acquisition by different species.

Another classification approach based on the developmental origin of roots (Cannon, 1949) was recently re-proposed by Zobel and Waisel (2010). This scheme is based on the distinction between a primary, secondary, and tertiary root system. The primary system consists of the primary (pole) root originating from the embryo radicle, basal roots originating from the hypocotyle/mesocotyle and lateral roots that emerge from these axes. The secondary system is built from shoot-borne roots and their laterals, while the tertiary system refers to fine roots below 0.6 mm diameter. Zobel and Waisel (2010) and Hochholdinger et al. (2004) demonstrated the distinct genetic control of these root types. Still when considering the entire root system, a developmental classification only results in a very general distinction between tap root dominated and shoot-borne root systems. This allows a differentiation between homorhizy in monocotyledonous and allorhizy in dicotyledonous species, while having limited capacity to capture more detailed distinctions within these groups.

A classification of root systems via their geometrical shape was suggested by Kutschera and Lichtenegger (1997). Based on extensive *in situ* observation of excavated root systems from over 1100 species, they distinguished between 11 fundamental rooting types from cord like (dominant vertical growth with few lateral extension) to discoidal (dominant surface near lateral extension).

A classification for woody species, combining morphological and functional attributes, was presented by Wahid (2000) who defined seven classes of different root foraging types.

These root system classification schemes presented so far differ in the traits they use for systematization and in the degree to which they capture taxonomic plant diversity. Traditional plant systematics used phylogenetic relationship to define species as a basic taxonomic unit with distinctive morphological attributes (e.g., Simpson, 2005). Fitter (1987) however questions the possibility to develop a species based root classification system and advocates a more functional approach to capture distinctive root system types.

Functional classifications have been used in plant ecology to define groups of species with shared biological characteristics that relate directly to function rather than phylogeny (Lavorel et al., 1997). Westoby and Leishman (1997) therefore speak of an ecological rather than a taxonomic classification. While functional/physiological traits can be used directly for classification (e.g., Wahid, 2000), the main idea is that the classification scheme has a functional rather than a phylogenetic meaning.

The idea of functional types rather than species as a classification unit seems particularly useful to be applied to root systems. Roots are morphologically less differentiated compared to shoots. Therefore, it is likely that species differences are often rather continuous than discrete regarding their root systems. Distinct, discontinuous groups of rooting types still might be found as a result of functional plant adaptation to different environments (e.g., Schenk and Jackson, 2002).

Functional classification schemes can be obtained using subjective, deductive and data-defined methods (Gitay and Noble, 1997). The subjective method derives functional groups based on field experience. It takes for granted that functional groups exist in an ecosystem and that they can be defined inductively by using plant attributes considered as essential grouping variables due to botanical expert knowledge and experience. The deductive approach uses an a priori established idea of important processes or properties in an ecosystem and deduces functional categories and related sets of traits from these premises. The data-defined approach uses multivariate statistics to seek for clusters that emerge from a set of attributes. The matrix of classification attributes for plant functional groups often depends on the objectives and context of a study. To obtain a generalized classification, some authors therefore suggested defining a core data set to extend the comparability between single studies (Weiher et al., 1999). Gitay and Noble (1997) recommended testing the uniqueness of any classification result for repeatability, using the same traits measured at different times or sites, for congruency, using different classification attributes, and for convergence, using data sets collected for different purposes.

The objective of this discussion paper is to present a functional classification of root systems. We show the shortcoming of single trait based comparative root research and suggest a new data-defined approach using multivariate statistics to derive functional classification of root systems. We hypothesize that such an approach can provide efficient classifiers to capture root system diversity. The main purpose of the paper is to encourage an exchange on root system classification and to suggest the use of multivariate methods as a way to obtain additional explanatory benefits from available root data sets in the frame of functional classification.

## Materials and methods

The classification scheme we suggest in this paper is first introduced by giving details on the applied statistical procedure. Then we present the data sets we use to exemplify the approach. Finally we suggest a core data set for classification and explain how our approach contributes to its elaboration.

### Statistical classification approach

The main purpose of the paper is to suggest a data-defined statistical classification method. We followed similar approaches used in ecology to obtain plant functional types, particularly the work of Gitay and Noble (1997). Our procedure is based on principal component analysis (PCA) and biplot inspection of root classification attributes, followed by cluster analysis using principal component based rooting types as composite classification variables.

PCA is a procedure to convert a multivariate dataset of various trait variables into few uncorrelated variables (principal components) that account for most of the variance existing in the original dataset. PCA is useful when some redundancy in measured variables can be assumed, i.e., mutual correlation among traits which are measuring the same construct. The extracted principal components optimally describe the common meaning of the single trait variables they contain. We used the SAS procedure PROC FACTOR to perform PCA. The number of principal components is determined based on the Kaiser criterion (Kaiser, 1960), retaining any component with an eigenvalue greater than 1.00. Also the increase in the explained proportion of variance by each further principal component is an indicator of how many components to keep. To improve the interpretability of principal components, different data transformations can be applied. A common way is to use rotation, i.e., a linear transformation of the factor solution. We used the orthogonal varimax rotation that results in uncorrelated principal components. Also other variable transformations can be useful to obtain principal components that better satisfy the interpretability criterion. The SAS procedure PROC PRINQUAL was used for this purpose.

An important result of PCA is the graphical representation of the solution via biplots. Biplots contain the single variables (i.e., root traits) as vectors and the single objects (i.e., species, cultivars) as points. The length of a vector is equal to the variance of the corresponding variable. A narrow angle between vectors of two variables indicates that they are strongly associated to each other. The origin of the biplot represents the average value for each variable. The more distant from the origin the projection of an object on a variable vector, the more this object deviates from the average in the variable. Inspection of biplots allows deciding which variables are essential to represent the overall variance of a studied object, i.e., which traits are necessary to obtain a comprehensive picture of a root system. This provides a way to ensure a high degree of congruency (i.e., same classification result is obtained with different sets of attributes) in the subsequent clustering. Also the repeatability of the classification result can be ensured when previously testing relations between the single variables over different years or sites.

The principal components as distinctive composite variables of different rooting types are then used in cluster analysis. Cluster analysis is a distance based method to identify common groups of species. This was done with the SAS procedure PROC CLUSTER. We notice here that for any statistical approach a possible influence of different mathematical clustering procedures on the uniqueness of the classification solution has to be taken into account (e.g., Milligan and Cooper, 1985; Lo Siou et al., 2011). Botanical expert knowledge is required when comparing the results of different clustering procedures that might not yield the same result as well as in the decision on the number of meaningful clusters. PROC CLUSTER however provides some statistical decision support on the number of clusters to retain (cubic clustering criterion, pseudo-*F*, pseudo-*t*^2^).

### Simulation of root system architectures

**Figure 1:**
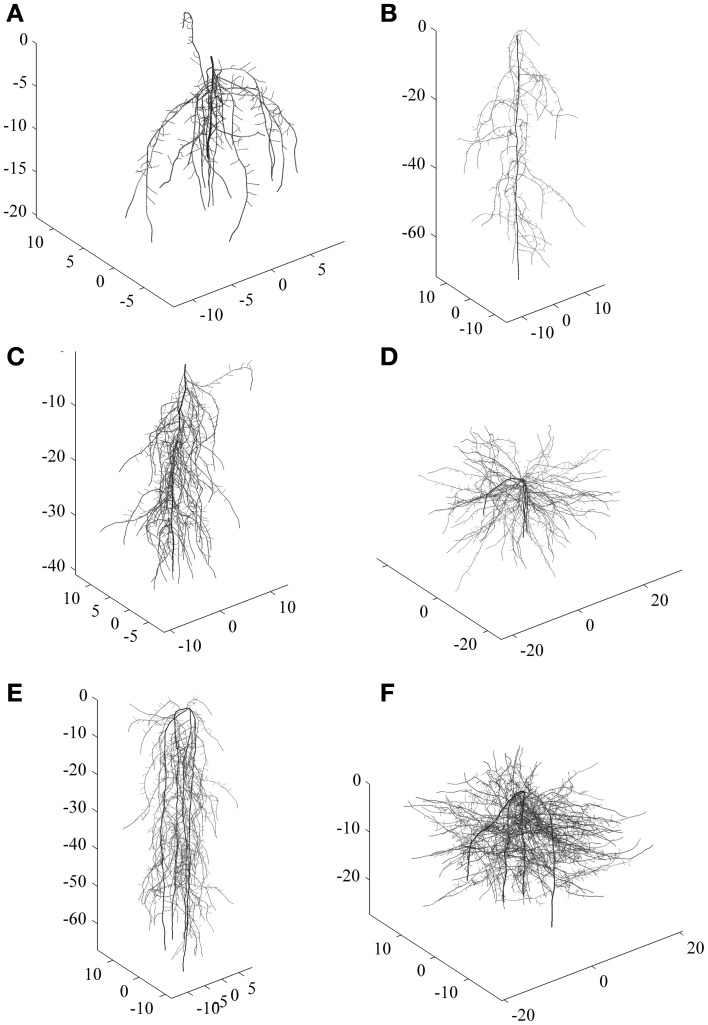
**Examples of simulated root systems corresponding to six main clusters. (A–C)** are tap root systems (one zero-order axes) differing in vertical root distribution and branching intensity, **(D–F)** are shoot-borne root systems (four zero-order axes) with different vertical distribution and branching intensity. **(A)** and **(B)** are more herringbone systems while **(D)** and **(F)** are typical dichotomous systems.

To exemplify the classification approach with a comprehensive root data set, we used a total number of 288 simulated root systems. They covered a wide range of diversity ranging from shallow laterally extending dichotomous root types to deep primary root dominated herringbone types. Some examples are shown in Figure 1.

For simulating the different root architectures we used the model of Leitner et al. (2010a). This Matlab based model uses L-systems (Prusinkiewicz and Lindenmayer, 1990) for the construction of branched root geometries. Basic production rules for root axes include root growth, root branching, and different types of tropism. Root axis elongation follows a negative exponential function as used by Pagès et al. (2004). Every root axis of a certain order produces lateral branches. Each axis is divided into three zones: the basal and apical zones near the base and the tip of the root, respectively, where no branches are produced, and the branching zone where new roots of successive order are created. Within the branching zone, a predetermined number of branches emerge. The spacing between the branches is determined by the section growth rule. The rule allows branches to occur at any point along the root axis and not only at segment edges with fixed segment length. The model allows simulation of different tropisms which cause root tip deflection. At the root system level both primary root dominated tap root systems as well as shoot-borne dominated fibrous root systems can be created via an adequate initial L-System string. In the case of a tap root system, the initial string consists of a single root tip of a zero order root. In the case of fibrous root systems, a number N_0_ of initial axes as well as the angle of their initial growth within a cone radius r_0_ is defined. Importantly the model includes a defined standard deviation for each parameter giving certain randomness in each simulation run even for the traits that were set by the user. For example the effect of soil particles on the direction of root growth is considered indirectly by some random variation of the growth direction.

Representation of real root architectures by the simulation model was originally validated by Leitner et al. (2010b) using digitalized images of excavated root systems of several plant species from the root atlas series by Kutschera (1960). Their study confirmed the proper biological basis of the parameter and production rules used by the model to simulate plant root systems.

The range of model parameters to simulate the diverse root architectures are given in Table 1 based on different literature sources.

**Table 1:** **Parameters and values used for simulating different root architectures**.

**Parameter**	**Values**	**References**
Number of zero-order axes	1, 4	Kutschera et al., 2009
Number of laterals per branching zone	20, 40, 60	Werner et al., 2001; Pasternak et al., 2005; Hund et al., 2009
Highest (4th order) lateral branching	Yes/no	Kutschera et al., 2009
Branching angle (zero-to-first order axes)	65°, 90°	Osmont et al., 2007; Nagel et al., 2009
Inter-branch distance at zero-order root	0.2, 0.5, 2.0 cm	Fitter, 1987; Arredondo and Johnson, 2011
Inter-branch distance at first-order root	0.1, 0.2 cm	–
Tropism type	Gravitropism, Exotropism	Rosen et al., 1999; Pagès et al., 2004

In our study the main objective of model application was to determine the key traits that shape root system diversity and to explore the most efficient classification variables among the various traits that make up the complex overall architecture of root systems. The results should also support the elaboration of a core data list and the development of targeted measurement strategies for root system classification.

For this model application some new evaluation tools were included into the code to obtain shape and topological root attributes for subsequent classification. Parameters obtained from the simulated root architectures were (i) macroscopic shape of lateral and vertical distribution according to Vrugt et al. (2001), (ii) topological branching parameters according to Fitter (1987) i.e., magnitude, altitude and external path length, and (iii) mean axes morphological traits (total root surface, average diameter, length per root order). These root attributes were then submitted to the statistical classification approach described in Statistical Classification Approach.

### Measured root system morphology

Application of the classification scheme to measured data was done using two samples of morphological traits from field experiments. Root traits were analyzed from soil cores (7 cm diameter) where roots were washed free from soil and then quantified by image analysis. Image analysis with WinRhizo followed the procedure described in Himmelbauer et al. (2004).

The first data set comprised morphological traits of different species used as cover crops for agro-environmental purposes. In this specific case root samples in the surface layer were taken to investigate root effects on soil structural properties in surface soil (Bodner et al., 2012). The sample comprised four fabaceae (*Vicia sativa* L., *Lathyrus sativus* L., *Trifolium alexandrinum* L., *Melilotus officinalis* L.) two brassicaceae (*Sinapis alba* L., *Raphanus sativus* var. *oleiformis* L.), one borginacea (*Phacelia tanacetifolium* Benth.), one linacea (*Linum usitatissimum* L.), one polygonacea (*Fagopyrum esculentum* Moench.), and one poacea (*Secale cereale* L.). Additionally two species mixtures were included (Mixture 1; *Secale cereale* L., *Trifolium incarnatum* L.*, Vicia villosa* Roth.; Mixture 2: *Phacelia tanacetifolium* Benth., *Sinapis alba* L., *Vicia sativa* L.).

Samples were taken from surface soil (2–7 cm soil depth) with two subsamples per plot (one sample on the plant row and one sample between rows) in three replicates. Although root parameters have been measured for a specific purpose in this study (influence on soil structure), the convergence criterion (Gitay and Noble, 1997) claims that the same classification should be obtained when the same species would have been sampled for a different purpose including other root traits. Beside basic morphological traits obtained by WinRhizo, we calculated root diameter ratio as an attribute describing lateral root extension. This was done by comparing root diameter in and between plant rows. The ratio between the two sampling positions indicated the change in diameter between branches near the primary axes (row) and more distant laterals (interrow). Furthermore, homogeneity between different root orders was approximated by the root diameter range, obtained by the coefficient of variation of root length in different diameter classes. A low diameter range means an even distribution of root length over all diameter classes.

The second data set originated from root samples of 12 cereal genotypes of different species and cultivars within a species. The data were obtained to determine differences in root systems relevant for drought resistance (Nakhforoosh et al., 2012). They comprised seven *T. turgidum* subsp. *durum* cultivars (Floradur and SZD3146 from Austria, Clovis from France, Matt from Arizona, 7060, 7063, and 7094 from CIMMYT, Mexico), two *T. monococcum* subsp. *monococcum* from Turkey (PI428154, PI428165), two *T. turgidum* subsp. *turanicum* (Kamut from Middle East, TRI5254 from Europe), and one *T. timopheevii* from Georgia (W9).

Four replicate soil cores were taken on the plant row. Samples from three soil depth (10–20 cm, 30–40 cm, 50–60 cm) were analyzed by WinRhizo. In this case depth distribution was calculated by the slope of a linear regression of root length densities vs. depths. Beside the morphological data also root capacitance (Chloupek, 1977) was measured and included in the analysis.

Measured data were first analyzed for each trait by univariate analysis of variance using SAS PROC MIXED. In case of repeated measurements over depths the mixed model was used with an AR(1) correlation structure for the repeated factor (Piepho et al., 2004). Subsequently, for those traits with significant differences at *p* < 0.05 mean comparison was done with a two-sided *t*-test. Thereafter multivariate analysis with PCA and clustering for root classification based on the morphological attributes was used as described above.

### Root trait core data set

The proposal for a preliminary root trait core dataset was derived from a literature survey. The objective was to estimate current availability of root data that could be used for classification. We did not pretend a comprehensive literature review, but a coarse overview of the frequency certain traits are measured. Therefore, we limited the survey to a keyword search in the Scopus database over the last 10 years. The number of hits in Scopus was used as indicator for the potential availability of datasets for the respective traits. A preliminary hierarchy among root trait was established based on the scale of observation. Different root attributes at each hierarchical level were listed and usual measurement methods indicated. In the frame of our classification approach biplot trait vector direction reveals which traits are essential to capture the overall diversity in a sample and thereby supports the elaboration of a core data set. Based on our simulation sample, covering several classes of root attributes, we show which class of traits is essential for a core data set according to our results.

## Results and discussion

The present discussion paper is motivated by the increasing need to understand root system diversity and functioning in several fundamental and applied research disciplines. For this purpose a common classification scheme is required to identify the distinctive root properties among different plant species. Here we suggest a data-defined procedure for functional root system classification and exemplify the approach with selected data sets.

### Univariate analysis restricts comparative root research

Comparative root studies have been done in ecology (e.g., Schenk and Jackson, 2005) and agronomy (e.g., Kashiwagi et al., 2005). Whenever large samples are involved, decision on the root parameter to use for comparison is strongly influenced by measurement constraints (e.g., Manschadi et al., 2007; Chloupek et al., 2010). In most cases it is uncertain if the selected trait is efficient to identify differences in the sample and captures the functional pattern of interest. The predominant method to decide on the differences within a sample is the application of univariate analysis of variance for each single trait.

**Figure 2:**
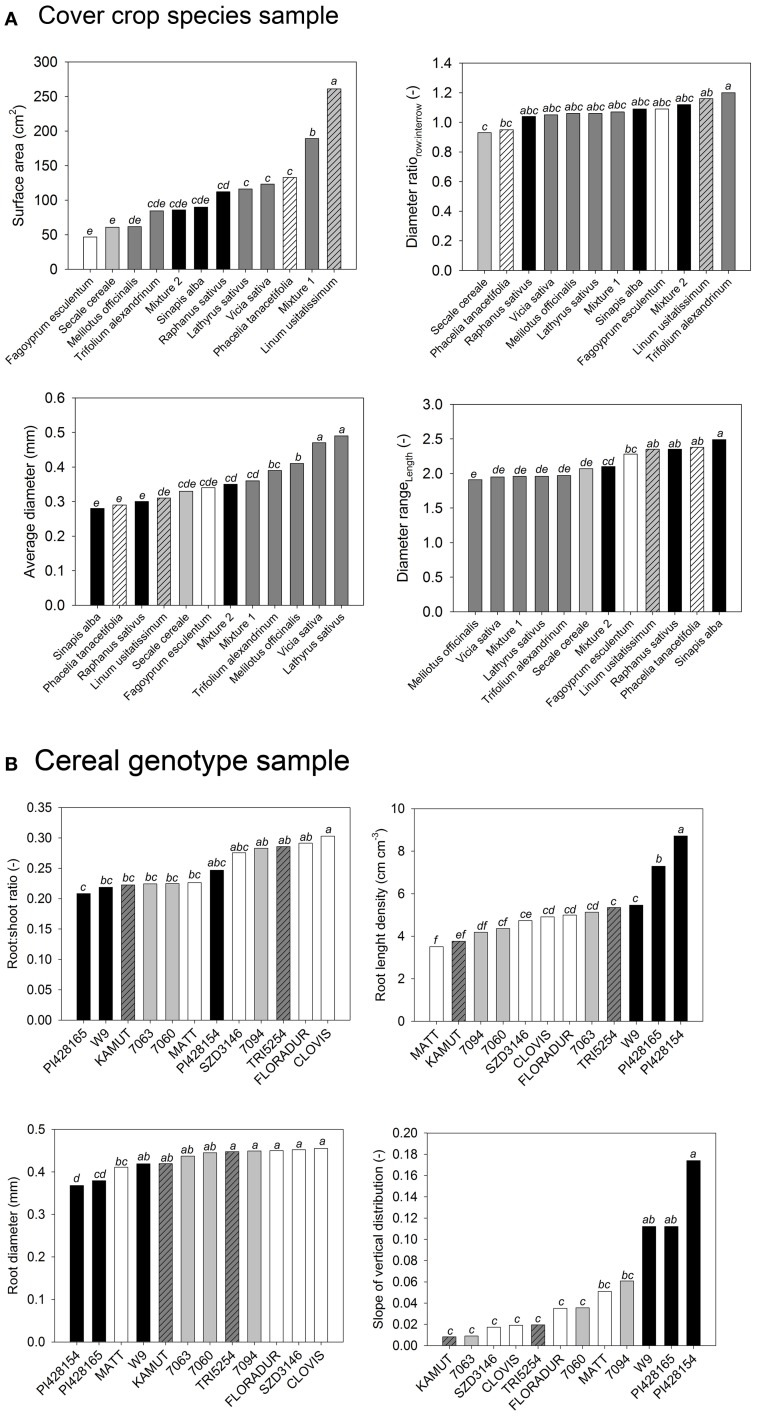
**Analysis of variance of selected root morphological traits from (A) the cover crop and (B) the cereal genotype sample**. Change in neighboring bars of species order shows that single trait analysis leads to different conclusions for each trait. (Statistical comparison of means is indicated by lowercase letters. Species sharing a common letter are not significantly different from each other at *p* < 0.05).

Figure 2 gives an example for this procedure.

The cover crop sample (Figure 2A) contained species from different plant families described by morphological root traits. Legume species were similar in root attributes related to diameter, while they differed in root surface area. They had a higher average diameter with less difference between row and inter-row sampling position (diameter ratio), and they had a more even root length distribution over different diameter classes (diameter range). On the contrary *Brassica* species were similar in total root surface area, but they differed in diameter related parameters. Similarity between *L. usitatissimum* and *P. tanacetifolia* could be expected from surface area, average diameter, and diameter range, while the two species clearly differed in the row-to-inter-row diameter ratio.

The cereal sample (Figure 2B) compares more closely related species of one family using root:shoot ratio, root length density, average diameter, and root depth distribution. The exotic genotypes (*T. monococcum* subsp. *monococcum, T. timopheevii*) represented extremes in most traits, in some cases (length density, depth distribution) with marked differences to the other genotypes, which however were not significant in all cases. The European *T. turgidum* subsp. *durum* genotypes were very similar in length and diameter, while showing different root:shoot ratio and depth distribution. Again their differences to neighboring genotypes (e.g., Mexican CIMMYT durum cultivars) were not clearly significant. Again the decision on distinctive groups of root systems within the sample and any interpretation of possible causes is hardly possible.

The following problems can be identified from these examples when relying on univariate analysis of variance for root system comparison:

- Ranking of species depends on the trait used. When using different traits, the conclusive picture remains unclear.
- Univariate analysis implicitly claims that traits are independent from each other, while they can be functionally linked. Separate evaluation of each trait therefore cannot provide a comprehensive comparison on the root system level.
- It is unlikely that a single root trait is sufficient to identify structural adaptation or functional behavior on its own. In most situations simultaneous consideration of more than one trait is required to understand adaptation and functioning (e.g., root response to soil compaction; Bengough et al., 2006).
- The outcome of single trait comparison is probably more study specific (different sites, different purposes) compared to multivariate approaches that capture the whole root system. This will easily lead to contradicting results and difficult inter-comparison between studies.

Comparative root research thus requires an approach that is more adapted to a multi-trait system which is hardly captured, neither structurally or functionally, by separate analysis of single attributes.

### Principal component based rooting types

To distinguish between different plant functional types, ecological research at the whole plant level combined morphological, physiological, phenological, and reproductive attributes (Duckworth et al., 2000). Fitter (1987) followed the general idea of functional classification and used a deductive approach based on the assumption that topological traits are most adequate to capture functional differences between root systems. Still it was not demonstrated that the topological classifiers most efficiently capture root system diversity compared to other root traits. In order to avoid any a priori decision on key classifiers we opted for a more open, data-defined approach via multivariate statistics (e.g., Kindscher and Wells, 1995; Naeem and Wright, 2003).

Following the plant functional type concept from ecology, we define rooting types, built from a distinctive combination of single traits, to be used as classifiers. Methodologically they are obtained by PCA, merging the single traits into independent composite variables (principal components). Hatcher (1994) underlines the importance of interpretability, i.e., the new variables should be biologically meaningful constructs. In a drought resistance study, Araus et al. (2007) demonstrated this by using principal components of several aboveground morphological attributes as classifiers for different regional origin of wheat species.

**Figure 3:**
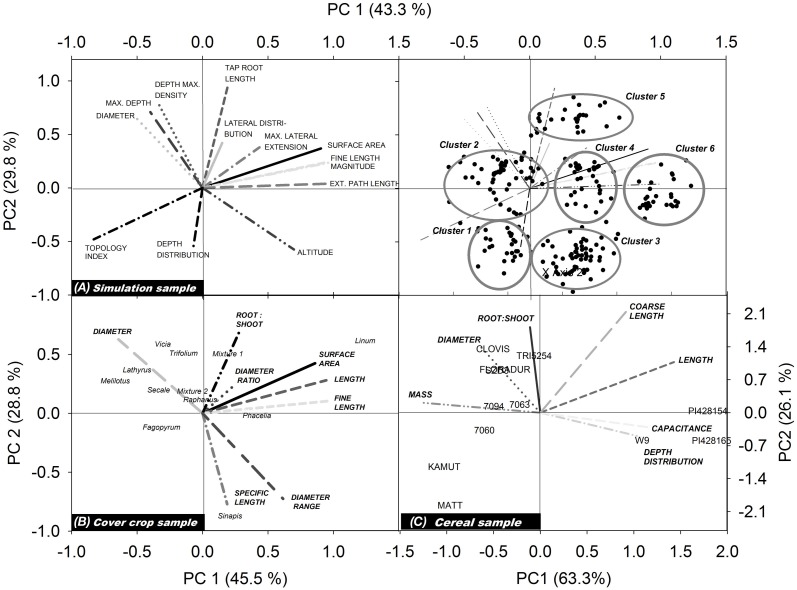
**Biplots showing trait vectors and location of the single objects from (A) the simulation sample, (B) the cover crop species sample, and (C) the cereal genotype sample**. For better visualization of the simulation results, trait vectors and objects are shown in separate biplots (for explanation of root traits, cf. section Measured Root System Morphology).

Figure 3 shows the biplots obtained from our three example data sets. Each biplot contains the root traits vectors and the location of the single species (objects) according to their principal component scores. For better visibility, trait vectors and objects for the simulation sample are shown on separate biplots.

For the simulated root systems the first principal component (PC1) captures density related attributes and Fitter's topological parameters, while the second principal component (PC2) is related to diameter and rooting depth mainly. According to the principal component scores, six distinct rooting types can be identified. Three groups are located along PC1, differing mainly in surface area, fine root length, and external path length. All these parameters capture the overall rooting density independent of shape or branching. Two further groups with a positive PC1 differed by their location along PC2, one containing roots with positive the other with negative PC2 scores. Thus, here vertical root distribution (tap root length, depth distribution) was the main distinction. The last group was located in the lower left quadrant with negative PC1 and PC2 scores. These systems are small sized herringbone roots with low branching and restricted depth penetration.

In the field sample with cover crop species of different plant families, trait vectors show a distinction between density dominated (high root:shoot ratio, high surface, length and fine length) rooting types vs. coarse (low specific root length) diameter dominated types. Root systems related to the density dominated rooting type are in the direction of a positive PC1, while those of the coarse diameter dominated rooting type are in the direction of a positive PC2. In the quadrants at the right side of the biplot (positive PC1) *L. usitatissmum*, *P. tanacetifolia* but also *S. alba* and the mustard-phacelia dominated mixture 1 are found. *Fabaceae* species are all found in the upper left quadrant, in the direction of the diameter vector and in the opposed direction of (fine) root length. High diameter/low density root systems shared by the *Fabaceae* species are probably related to root-microbial interactions which are characteristic for this plant family. The high root diameter of legumes is supposed to have evolved for properly hosting their root symbionts (Eissenstat, 1992).

In the cereal genotype sample the two fundamental rooting types are also related to high (positive PC1) and high diameter/mass (positive PC2). Here also depth distribution is contained in PC1, with surface near root systems at the right side of the biplot (positive PC1) and deep rooted species at the left side (negative PC1). This indicates a trade-off between high resource exploitation potential by dense root systems and strong exploration capacity of deep rooting types (Fitter et al., 1991; Fitter, 2002). Wild genotypes (*T. monococcum* subsp. *monococcum, T. timopheevii*) are located on the right hand of the biplot (positive PC1), particularly in the lower quadrant. Thus, they are characterized by root systems of high density with a surface near concentration of root axes. *T. turgidum* subsp. *durum* cultivar Matt and the *T. turgidum* subsp. *turanicum* variety Kamut are both located in the lower left quadrant, sharing a high negative value of PC2. They show a rooting type with below average density, and high root allocation to deep soil layers. The central European genotypes are all located in the upper left quadrant with high PC2 and negative PC1, i.e., showing a deep rooting, diameter dominated rooting type. CIMMYT genotypes are found around the biplot origin suggesting an intermediate rooting type.

In spite of the different number and type of root traits in each data set, in all cases there was a high influence of rooting density traits (e.g., length, fine length, and surface area) on the first principal component, while the second principal component was always positively related to high average diameter and dominance of coarse root axes. The common meaning underlying the principal components is demonstrated quantitatively by a significant relation of principal component scores of common root traits between the different samples (simulation vs. cover crop sample: *r*^2^ = 0.65, *p* = 0.02^*^; simulation vs. cereal sample: *r*^2^ = 0.82, *p* = 0.002^**^; cover crop vs. cereal sample: *r*^2^ = 0.65, *p* 0.05^*^). This proves that the principal components indeed provided meaningful constructs expressing distinct rooting types.

In our examples principal component based rooting types captured between 74% (cover crop sample) and 89% (cereal sample) of the overall variability. A third principal component would have increased the explained variance between 6.6% (simulated sample) and 14.5% (cover crop sample) and might be considered for some cases.

### Functional classification based on rooting types

Our data-defined approach intends to build functional classification from principal component based rooting types integrating all available root trait information. Each species is characterized quantitatively by principal component scores that identify its association with the distinct rooting types.

**Figure 4:**
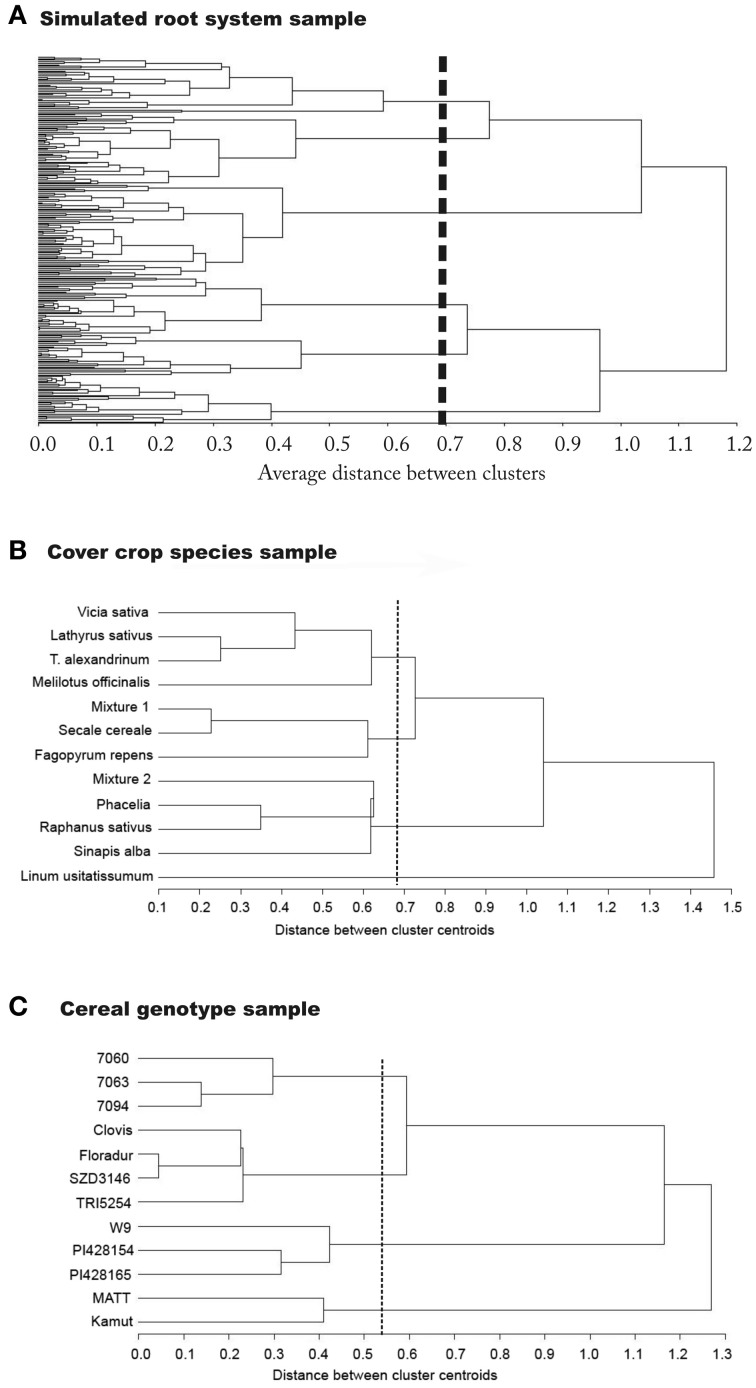
**Dendrograms showing the classification result from principal component based rooting types used as classifiers**. Results are from **(A)** the simulation sample, **(B)** the cover crop species sample, and **(C)** the cereal genotype sample.

The classification step is then done using cluster analysis. This statistical method determines the distance between the species contained in a sample. The resulting dendrograms illustrate the number of functional groups that emerge from the data, containing all species with similar rooting types. The emerging groups are an adequate functional classification unit to be used for further interpretation of underlying causes (e.g., genetic relationship, environmental adaptation). Figure 4 shows the dendrograms calculated from our three example data sets.

For the simulated root sample, statistical criteria (cubic clustering criterion, pseudo-*F* and pseudo-*t*^2^) suggests six groups to be retained in the dendrogram. There are two clearly distinctive clusters with highest average distance. These are related to the number of zero order axes (one vs. four zero-order roots). However, the total number of clusters with distinctive groups to be considered in the data-set is six. These groups differ by (i) the number of zero order axes, (ii) the number of lateral branches, and (iii) the inter-branch distance between laterals along the zero-order axis. The three first clusters from top to bottom along the dashed line of the dendrogram all represent tap-root systems with increasing number of lateral branches. Inter-branch distance on the tap root is similar in cluster one and three, while cluster two had around twice the length between branches. Clusters four to six contain systems with four zero-order roots (approximation of shoot-borne root systems) again with increasing number of lateral branches from four to six. Inter-branch distance on the zero-order roots is highest in cluster five and lowest in cluster six.

Taking into account the root traits contained in the principal component based rooting types, clusters one, two, and six mainly differ due to their rooting density (PC1 score), while for clusters three to five the main distinctive criterion is their depth distribution (PC2 score). Considering root topology as classifier, clusters one and two fall in the category of low branched herringbone systems, while cluster six represents a strongly branched dichotomous system.

Using shape and morphological indicators only, four distinctive clusters would have been retained. The same number of cluster would have been obtained when only considering topological indicators. When reducing classification variables to single axes morphological attributes (diameter, surface area, fine lengths) only, three distinctive groups would have been identified (dendrograms not shown).

The classification results from the simulated root data show that developmental type (branching from a single zero-order axis originating from the embryonic radicle vs. branching from several zero-order axes originating from shoot nodes) is indeed a fundamental distinctive criterion at a high hierarchical level. This is in agreement with the recent developmental classification of Zobel and Waisel (2010) showing the decisive role of root origin to understand different root system structures. Several morphological properties related to rooting density strongly depend from developmental type. Still the results also highlight our criticism on developmental classifications. They are restricted to a very coarse distinction (tap vs. shoot-borne systems) and insufficient to reveal all existing groups with distinctive rooting types.

To extract all six distinctive groups identified by statistical indicators, density related, shape related (distribution), and topological classifiers are required. Topological classification parameters from Fitter (1987) and morphological attributes with macroscopic shape parameters are equally efficient classifiers when used separately (four clusters). Still a disadvantage of the topological approach is the difficult measurement of topological branching structure. This is reflected by the low number of studies reporting this type of data (cf. Table 2). With quickly increasing *in situ* imaging possibilities however we might expect better architectural data for topological classification in future (Zhu et al., 2011; Ingram et al., 2012).

**Table 2:** **Traits at different observation scales for a comprehensive, hierarchically ordered core data set to classify root systems, common measurement methods, and Scopus database hits from keyword search**.

**Hierarchical level**	**Parameter**	**Method**	**Hits**
Whole plant traits	Root biomass	Dry weight	2166
	Root: shoot ratio		1165
Root system shape	Maximum rooting depth	Excavation	27
	Maximum lateral extension	Curve fitting to morphological data	1
	Depth distribution		303
	Lateral distribution		41
Root developmental traits	Number of seminalroots	Root observation on young plants (e.g., gel chambers, blotting paper)	53
	Emergence of shoot-borne roots		18
	Initiation of lateral branching		13
	Maximum number of lateral branching orders		8
Root branching traits	Average branching angle	2D and 3D *in situ* observation and image analysis	10
	Distance between lateral branches		2
	Link length (internal, external)		4
	Topology (magnitude, external path length, altitude)		23
Axes morphology	Root length (surface) density	Destructive sampling (soil cores) and image analysis	473
	Average root diameter		550
	Root length/surface per diameter class		90
	Decrease of root diameter per root order		13
	Specific root length		249
Root anatomical and physiological traits	Xylem vessel number and diameter	Root anatomical cuts and microscopic measurement	153
	Cortex thickness		51
	Suberization/lignification	CO_2_-flux	36
	Root respiration		422
	Aquaporin abundance	PCR	308

On the contrary adequate spatial sampling schemes for morphological traits (Bengough et al., 2000) to derive shape parameters as used here (Vrugt et al., 2001) provide more easily available classifiers that also capture most of the diversity resulting in a meaningful classification. Axes morphology and macroscopic distribution seem to be a reasonable compromise between coarse distinction due to development type and the fine differentiation that requires topological branching infotmation.

For the cover crop species data set, four groups are suggested by the cubic clustering criterion. The dendrogram clearly separates legumes at one end, while *L. usitatissmum* forms a separate group at the opposite side. The density dominated rooting types (*Brassicaceae*, *P. tanacetifolia*, mixture 1) form a common group, while *S. cereale*, *F. esculentum*, and the legume-rye mixture 2 are an intermediate group between the diameter and density dominated rooting types. The distinction between the large diameter dominated legume systems and the density dominated *Brassica* systems can be interpreted functionally as being associated either with strong exploitative potential (fine roots, high root-soil contact surface) or high explorative capacity (Fitter, 2002), in the case of legumes also influenced by rhizobia colonization.

At the taxonomic order of plant family the cover crop species data revealed that only for certain families a distinctive rooting pattern could be identified. *Fabaceae* clearly clustered together sharing a high diameter/low density rooting type (high PC2). Also *Brassica* species were in a joint cluster (density type with positive PC1). However, they shared their cluster with species from other families. This suggests that either morphological descriptors are insufficient to capture differences in the root systems of these plant families, or that indeed these species, used as autumn grown cover crops, have similar rooting types due to similar environmental adaptation in spite of belonging to different families.

The cereal genotypes fall into four distinct functional groups. Mexican CIMMYT durum genotypes are grouped at one end of the tree, while Matt and Kamut are located at the other end. The latter form a low density deep rooting type (low PC1 and low PC2), while the CIMMYT genotypes are an intermediate type (PC1 and PC2 near origin). A separate group is formed by *T. monococcum* subsp. *monococcum* and *T. timopheevii*, characterized by a density dominated shallow rooting type (high PC1). A fourth cluster contains Central European durum cultivars as well as the European *T. turgidum* subsp. *turanicum* genotype with high diameter rooting types of intermediate density and depth (high PC2 and PC1 near origin).

The cereal data set shows the relation between regional origin and rooting type. Genotypes from summer dry climates (Arizona *T. turgidum* subsp. *durum* var. Matt and Middle Eastern *T. turgidum* subsp. *turanicum* var. Kamut) share a common cluster, different from all others, in spite of belonging to different subspecies. Also *T. turgidum* subsp. *turanicum* var. TRI5254 with unspecified European origin falls in a common cluster with the Central European durum species, which seems to be related to regional origin. In case of the exotic genotypes (*T. timopheevii* and *T. monococcum* subsp. *monococcum*), sharing a common cluster of different sub-species too, probably low breeding intensity is the main reason for their similar root systems (Reynolds et al., 2007). Their overall habitus rather resemble natural grass species than modern cereal crops. The cluster formed by CIMMYT durum cultivars could be expected as they have a common genetic background. Although regional origin is important in many cases, still similarity in the genome seems to be a basic factor underlying the joint clusters: *T. turgidum* subsp. *turanicum* and subsp. *durum* both have genome BA^u^, while *T. monococcum* (A^m^) and *T. timopheevii* (GA^m^) both contain the A^m^ genome (originating from *T. uratu*).

The classification results from the two field samples confirms that meaningful groups can be extracted based on principal component based rooting types. In most cases possible causes for common groups of species could be identified. Distinctive groups showed a relation to the genetic background, suggesting that also at the level of root systems, similarity due to phylogenetic relation can be expected. However, also common environmental adaptation and breeding background seem to be relevant causes for root system formation. In some cases a more accurate distinction might be obtained when topological root attributes would be included. However, the various potential causes for common clusters also underlines the idea of a functional classification where environmental driving forces might have a dominant impact on the rooting type beyond phylogenetic relationship.

### A core data set for classification

We have shown that PCA provides meaningful and efficient classifiers that integrate all available root information without a priori deciding on their importance for comparative classification. Some authors working on functional classifications suggested core data sets to improve the comparability among classification results (Weiher et al., 1999). Optimally the traits in a root core data set should fulfill three main criteria: (i) they should provide a biologically meaningful and comprehensive root system description; (ii) they should contain traits that efficiently distinguish between root systems and capture as much detail of species differences as possible; (iii) it should comprise traits that are readily measureable with agreed protocols.

Table 2 suggests a list of root core traits covering different structural and functional scales of a root system. The hierarchical order of traits assumes that discrete classes of root systems can be found on higher scale (overall root system size and shape, developmental type), while at lower scale (single axes morphology, physiological functioning) differences are more continuous. However, important exceptions from this rule may exist (e.g., strongly distinctive anatomical attributes). We also give related measurement methods and trait availability via the number of hits from a Scopus database search. Database review clearly shows that root information is mainly available at the level of total root biomass (27% of studies). Also single axes morphology (8%) and some root physiological traits (e.g., respiration, aquaporines; 6%) were reported more frequently. On the contrary architectural traits are rarely measured, particularly in mature root systems, and some relevant physiological parameters (e.g., root resistances) were absent at all in our database screening.

Reich (1993) suggests that the number of traits required for a minimum data set can be reduced by statistical methods exploring the covariance between traits. Within our data-defined classification approach, this can be done previously to clustering during biplot inspection. When trait vectors have a similar direction, they show joint variability. In this case some descriptors can be substituted by others (e.g., more easily measureable traits), without losing information on the whole system diversity and ensuring the congruency of a subsequent classification result (cf. Appendix). Also trait stability and thereby expected repeatability of the classification result can be assessed via biplot inspection in case of multi-location or multi-year data sets. While for example root diameter is a rather stable trait, root distribution and root-to-shoot ratio strongly respond to different environmental conditions (cf. Appendix). For traits strongly responding to different environmental conditions, a plasticity index (e.g., Bell and Sultan, 1999; Valladares et al., 2006) could be included to captures root-soil interaction effects on root system structure.

Also root architecture models could be an important tool to support elaboration of a core trait list for classification. To our knowledge after the publication of Fitter (1987) and his successive studies, we are the first in applying a root architecture model in the context of classification. The main intention was to determine strong classifiers to distinguish different root systems within the architectural diversity to be expected in nature. Our results suggest that information on axes morphology, root system shape and branching (topology) is required to capture all distinctive groups within a large sample. The lack of architectural measurement data can only partially be covered by other root attributes such as macroscopic shape descriptors.

Classification results presented in this study were limited to differences in root structural attributes rather than functionality (e.g., water or nutrient uptake). When including functionality, other traits (e.g., branching angles, tropisms) could become more important for capturing distinctive rooting types. The relation among a classification based on structural vs. strictly functional/physiological attributes could be studied theoretically when calculating functional differences resulting from the different root architectures simulated here.

Although there is still a lack of certain type of root data (e.g., mature root system architecture), the exponential increase in root studies since the 1960s strongly suggests that a general classification of root systems it is not mainly a problem of lacking data, but rather of an agreed effort and method to join existing knowledge. An open data-define classification scheme presented here could contribute to close this gap in root research.

## Conclusions

This paper presents a data-defined approach for classification of root systems. The objective is to provide a frame to identify the main distinctive root system properties among different species, analyze potential driving forces in the evolution of root system diversity, and study the functional implication of different rooting strategies. We follow the concept of “plant functional types” used in ecology and ecophysiology to search for common groups on a functional rather than a phylogenetic basis. We propose a data-defined approach where distinctive groups are obtained via statistical data exploration methods without a prior decision on a selected classifier.

Our main conclusions are that root morphological description with adequate spatial data provides reliable attributes to classify different types of root systems. This was shown by both simulated and field measured root data that allowed identification of main common rooting types based on average axes morphological attributes. Still a more accurate classification could be expected when integrating topological information. Root system types are the joint result of phylogenetic relation and environmental as well as human selection pressure. A functionally based classification is therefore most appropriate to capture diversity among root systems. The data-defined approach can integrate the increasing number of knowledge on root structure and functioning. PCA and biplot based data inspection provide methods to determine key traits for a core data set and ensures a high degree of stability in cluster based grouping.

The data-defined classification method encourages integration of results from different measurement scales and research foci to capture the overall root system diversity for a broad classification scheme. Currently numerous information on average axes morphology and spatial distribution is available as a result of efficient image analysis tools. Our study suggests that such morphological data sets (length, diameter, depth distribution) would constitute a reliable initial step for classification beyond the established coarse distinction based on developmental type. For a detailed classification of root functional types however a core data set requires quantitative knowledge on root branching. Future integration of root functionality will further extend the basis of our approach and highlight the role of functional vs. structural attributes for classification of root functional types.

### Conflict of interest statement

The authors declare that the research was conducted in the absence of any commercial or financial relationships that could be construed as a potential conflict of interest.

## Figures and Tables

**Figure A1:**
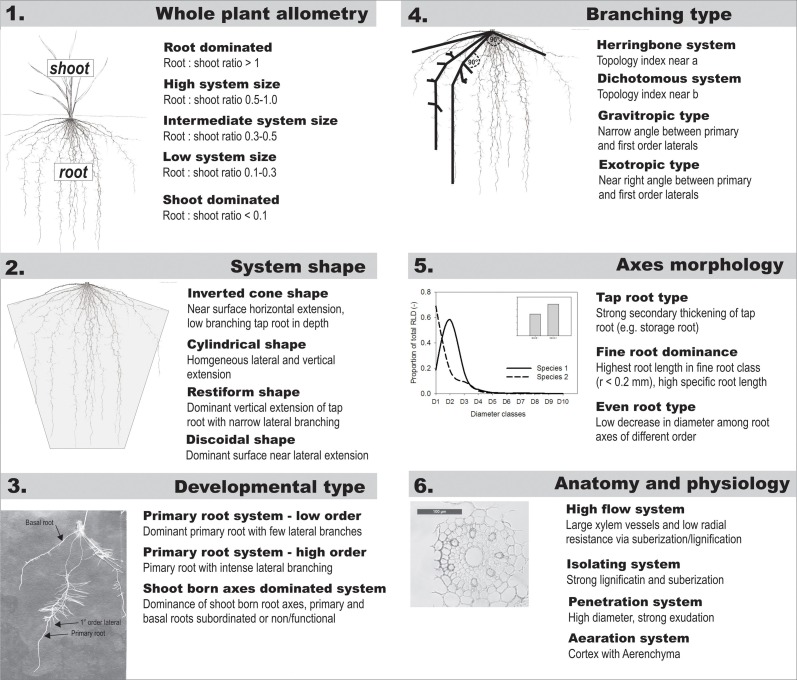
**Examples of possible descriptive categories for hierarchically ordered root traits within a comprehensive core data set to be used for classification of root system types**.

**Figure A2:**
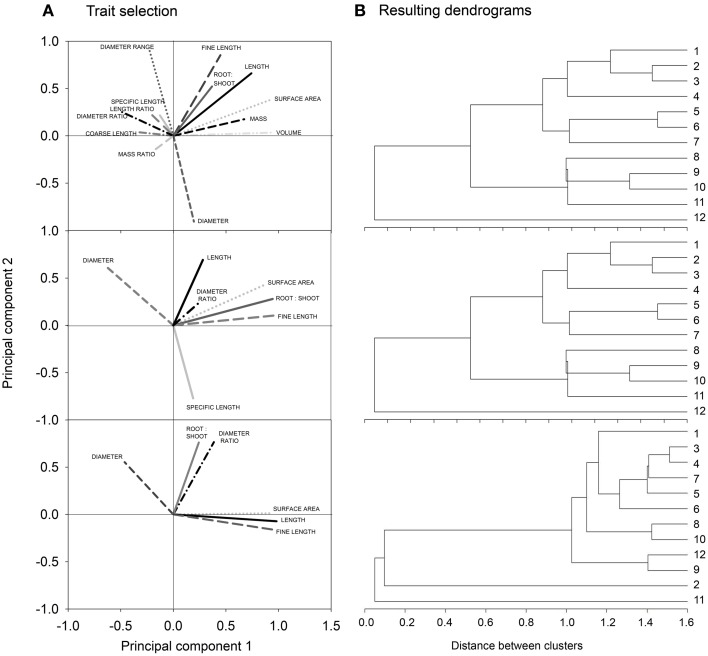
**Example for biplot inspection (A) to ensure congruency of subsequent grouping via cluster analysis (B)**. Biplots are used to visualize the mutual relation between different traits and to determine which traits are essential to conserve the overall variance in a multi-trait root system characterization (Data from species experiment, cf. Measured Root System Morphology; Root:shoot is root-to-shoot dry matter ratio; mass, length and diameter ratio are indicators of lateral distribution calculated by dividing the respective parameter measured in and between plant rows).

**Figure A3:**
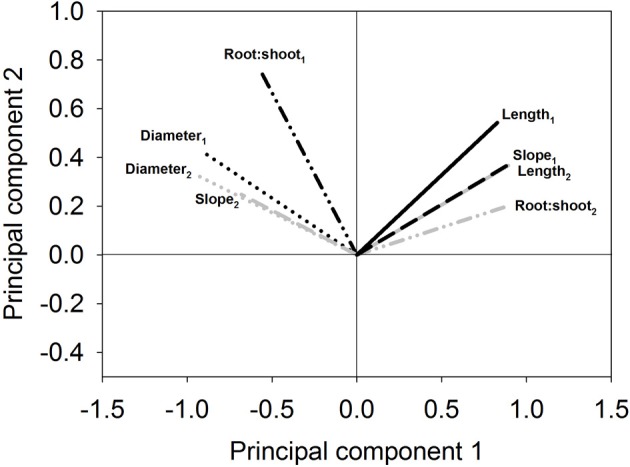
**Example of biplot to check expectable repeatability of groups in subsequent clustering**. Measured morphological traits of the same genotypes at the same site over 2 years. (Subscripts indicate first and second year sampling; data from *Triticum* experiment; slope is calculated from the root length density decrease over soil depth).

## Footnotes

^1^According to Troll (1943) the term “radication” would be more appropriate then the term “root system.” He defined a root system to consist of branches from a single root (e.g., from the pole-root or a single shoot-borne root). Following this definition, all shoot-borne roots of monocots form distinctive systems, while the term radication comprises all of these root systems of a plant. However, as most root studies use the term “root system” instead of radication, we also make use of this term.

## Appendix

In Table 1 we suggested a core set of root attributes that provide a comprehensive description of the root system. Figure A1 gives an example of root classifiers at different hierarchical levels. Qualitative descriptors can be derived for each attribute, resulting in a composite descriptive characterization of a given rooting type.

An initial distinction is made from whole plant allometric relations such as root:shoot ratio which capture assimilate allocation between aboveground and belowground organs. Following Kutschera and Lichtenegger (1997) a major distinction among root system types is expected from the overall root system shape which can be defined by a geometrical form. The geometrical form or spatial distribution is influenced by developmental type (dominance of the primary root, number of basal roots, extensiveness of shoot-borne roots) as well as the topological connection between axes. These classifiers are thus placed on the next hierarchical orders. The morphological description of single root axes via average descriptors or derived attributes (e.g., diameter or frequency distributions) logically follows on the subsequent level. Finally root anatomical and physiological characteristics are set at the basis of the hierarchical scheme. An example of root classification attributes and distinctive categories at each hierarchical level is shown in the Appendix.

For a stable classification result, it is essential to test the root attributes used. Within the data-defined approach we presented, this can be done at the step of biplot inspection. Particularly biplot analysis serves to investigate the relation among traits used as classifiers. Knowledge of collinear relations between data is important to ensure the congruency of classification in case of using different classifiers. This is exemplified in Figure A2. Vectors of similar direction express joint variability of traits, while a perpendicular direction shows traits not related to each other (Figure A2A). When using principal components for clustering, dendrograms remain stable as long as all main vector directions in the biplot are covered by traits. Thereby the description of the overall variability of the system is conserved (Figure A2B). Thus, traits showing similar vector direction might be substituted by one another.

Also stability over years or sites (repeatability of classification) can be assessed in this way (Figure A3). It can be seen that two of the traits (root:shoot, slope of depth distribution) have a roughly perpendicular angle between their vectors. Also the length of the vector is different. Thus, these traits are very sensitive to environmental conditions (years, sites). Root diameter and root length on the contrary show similar vector directions and length revealing their stability over the 2 years. The latter traits therefore can be expected to result in higher repeatability when used for cluster based classification.
